# Polio vaccination activities in conflict-affected areas

**DOI:** 10.1080/21645515.2023.2237390

**Published:** 2023-07-25

**Authors:** Chukwuma Mbaeyi

**Affiliations:** Global Immunization Division, U.S. Centers for Disease Control and Prevention, Atlanta, GA, USA

**Keywords:** Polio, poliovirus, vaccination, conflict, insecurity, outbreak, war, immunization

## Abstract

Conflict poses a threat to the stability of health-care systems around the world. Within the context of immunization service delivery, conflict-affected geographies are often dogged by recurrent disease outbreaks due to the inability to administer life-saving vaccines to children residing in these areas. Essential immunization coverage is often poor in conflict-affected geographies, and within the specific context of the Global Polio Eradication Initiative (GPEI), multiple rounds of supplementary immunization activities are often needed to compensate for the inability to provide adequate immunization services. In order to implement polio vaccination activities, GPEI has often resorted to innovative approaches to reach and vaccinate children in security-compromised areas. This article examines the approaches adopted by the global polio program in conducting vaccination activities in conflict-affected geographies with the aim of understanding how they have influenced the successes and setbacks of the program in its bid to eradicate all polioviruses.

## Background

The rise in political conflicts in the past decade poses a significant threat to the stability of health-care systems around the world. The delivery of essential health-care services, including the administration of life-saving vaccines, has been severely compromised in areas affected by war and humanitarian disasters.^1–4^ Some of these conflicts, as in those in Afghanistan and Somalia, have been protracted,^5–7^ resulting in persistent deficiencies in the quality of healthcare service delivery over several decades. Others have seen the sudden outbreak of war lead to destabilizing effects on health-care systems that were otherwise stable and well-functioning.^4,8,9^ Compounding the impact of conflict on national and local health-care systems is the occurrence of multiple communicable disease outbreaks,^10–12^ which often take place in the setting of large-scale humanitarian displacement within and across national borders.^13^ Due to the depletion of health-care resources in systems that are concomitantly overwhelmed by multiple emergencies, the delivery of essential immunization services is often compromised. Consequently, populations in conflict-affected geographic areas tend to rely on supplementary immunization activities (SIAs) provided through ad-hoc humanitarian services or well-established public health programs, such as the Global Polio Eradication Initiative (GPEI), to compensate for the inability to provide adequate essential immunization services.^14,15^

GPEI, established in 1988 by the World Health Assembly,^16^ is a global partnership of health ministries around the world with leading international organizations, including the World Health Organization, Rotary International, the U.S. Centers for Disease Control and Prevention, UNICEF, and, more recently, GAVI (the Vaccine Alliance), and the Bill and Melinda Gates Foundation. The initiative is aimed at the eradication of poliomyelitis, a viral disease capable of causing paralysis and death.^17^ Since its inception, GPEI-led vaccination activities have resulted in a > 99.9% reduction in the number of polio cases globally. Only two countries (Afghanistan and Pakistan) remain endemic for transmission of wild poliovirus (WPV) type 1, the only wild poliovirus serotype still in circulation.^15,18^

These remarkable results have been achieved in spite of the obstacles faced by the global polio eradication program over the course of its existence. GPEI has often had to navigate the treacherous path of conflict, chaos, and political instability to bring life-saving polio vaccines to children in all parts of the world. Financial investments worth billions of dollars and the dedicated service of thousands of frontline polio workers^19,20^ over more than three decades have enabled the program to reach millions of children and families in war-torn and security-compromised areas, including during periods of active military combat and militant insurgency.^21^ In some countries, like Pakistan and Nigeria, conflicts and security challenges are mostly confined to specific regions^22–24^ that constitute priority areas for the polio program. In countries like Afghanistan and Somalia, with weak and unstable central governments, security and access issues are often more dispersed across most of the country.^5,6^ Conducting polio vaccination activities in these countries pose significant challenges not only because of intercurrent conflict but also because of weak immunization delivery systems. Consequently, GPEI relies heavily on the implementation of repeated rounds of supplementary immunization activities in each of these four countries^25^ and other similarly affected countries in order to compensate for underperforming routine immunization systems and raise polio immunity levels sufficiently among eligible children.

Supplementary immunization activities have been pivotal to GPEI’s success in reducing the number of countries with endemic wild poliovirus transmission and have been crucial in responding to outbreaks of both wild and vaccine-derived polioviruses in politically unstable and conflict-affected areas.^26,27^ Polio outbreaks spreading from reservoir countries into once polio-free countries have been and remain a recurring and recalcitrant threat to the progress achieved by GPEI. Within the context of armed conflict, the emergence of these outbreaks is often driven by the destabilizing effects of war on the health-care systems of previously stable countries or the persistence of conflict, which compromises security and access for the conduct of vaccination activities. Strategic responses to these outbreaks, primarily utilizing oral poliovirus vaccine (OPV), have yielded varied results. Responses in Syria and Iraq resulted in successful interruption of outbreak poliovirus transmission,^14,28,29^ whereas results in places like the Democratic Republic of Congo and the Horn of Africa have been mixed,^30–32^ with initial successes undercut by subsequent setbacks. Conflict, while a driver of polio outbreaks and a hindrance to responses in each of these countries, has been surmountable to varying extents. Further, it has not necessarily been the most important predictor of the effectiveness of response activities. Organizational capacity, political commitment, community attitudes, and historical vaccination coverage have been crucial to the success of polio vaccination activities in settings of conflict.

This article examines the implementation of polio vaccination activities in settings of war and other armed conflict with an aim to understand the approaches that have been adopted in these settings and how they have influenced the successes and failures of the global polio eradication program.

## Methods

Literature indexed by the National Library of Medicine on polio vaccination in conflict-affected areas were reviewed using a variety of approaches. Using search teams such as “Polio Vaccination AND War AND Conflict” in PubMed (https://pubmed.ncbi.nlm.nih.gov/), 36 articles addressing the specific issue of polio vaccination activities in conflict-affected areas, considering the period during 2000–2022, were identified. To broaden the scope of the search, more generic search terms such as “Polio Outbreaks” and “Polio Vaccination” were used to identify articles germane to the review that may not have been unearthed by the more restrictive search terms. Additionally, annual Morbidity and Mortality Weekly Reports (MMWRs; https://www.cdc.gov/mmwr/polio_reports.html) on polio eradication since 2012 were reviewed, with a specific focus on global program update reports, progress reports for endemic countries, and outbreak response reports.

Findings were organized into two broad categories: (a) polio vaccination in endemic countries with a specific focus on activities in conflict-affected areas and (b) outbreak vaccination activities within the context of war and insecurity or political instability. Within each of these categories, I provided an overall context for polio eradication activities; described the nature of extant conflict or insecurity and its impact on polio activities; identified approaches and strategies adopted for the conduct of vaccination activities, including the scope of these activities and the results achieved; and highlighted ongoing risks and challenges to polio vaccination activities in these areas.

## Findings and discussion

### Polio-endemic areas (Figure 1)

In the past decade, Afghanistan, Pakistan, and, until 2016, Nigeria were endemic for wild poliovirus transmission.^15,33^ They have also experienced multiple outbreaks of circulating vaccine-derived poliovirus (cVDPV).^34,35^ In these countries, six or more polio SIAs are conducted annually using a variety of polio vaccines, including trivalent OPV (tOPV; types 1, 2, and 3), bivalent OPV (bOPV; types 1 and 3), monovalent OPV (mostly type 2, i.e., mOPV2), and inactivated poliovirus vaccine (IPV).^36–38^ Up until 2016, when tOPV was withdrawn from the immunization schedules of all countries,^39^ most SIAs were conducted using either tOPV or bOPV nationally or sub-nationally. The scope of vaccination activities in these countries is usually informed by a prioritization scheme, where areas deemed at highest risk of poliovirus transmission or with active ongoing circulation are more frequently covered through SIAs compared with other areas of the country. National immunization days (NIDs) encompass all of the country and target all eligible children under 5 y of age, often tens of millions of children, whereas sub-national immunization days (SNIDs) cover only limited areas of the country, prioritized by risk profile and typically targeting children under 5 y of age.^25^

**Figure 1.**
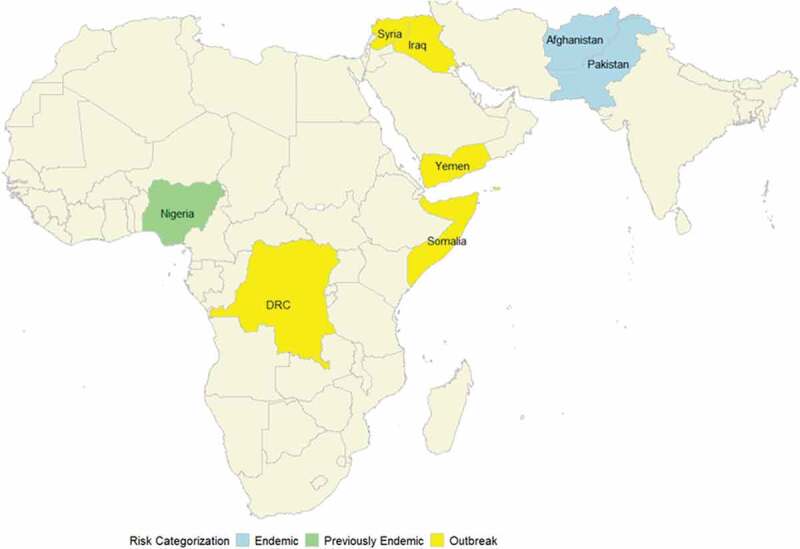
Map showing selected conflicted-affected countries with poliovirus transmission based on wild poliovirus endemicity and polio outbreak status during 2012–2022.

Conflict and insecurity have exerted substantial impact in altering the scope and quality of both NIDs and SNIDs in polio-endemic countries. In Afghanistan, coverage in the southern provinces of Helmand and Kandahar, as well as in several of the eastern and southeastern provinces, including Kunar and Nangarhar, has been severely impacted by protracted conflict, resulting in several parts of these provinces being partially or entirely inaccessible for polio SIAs on an intermittent or prolonged basis.^38,40,41^ In January 2020, as many as 2.75 million children <5 y of age were inaccessible for polio vaccination during SIAs.^38^ In Pakistan, significant security challenges in the southern districts of Khyber Pakhtunkhwa have resulted in a chronic, intractable problem of inaccessibility, in which tens of thousands of eligible children are repeatedly missed during polio vaccination activities.^42,43^ The picture is somewhat similar in Nigeria, where vaccination activities in northeastern Nigeria, predominantly in Borno and Yobe states, have been disrupted by the activities of extremists and insurgent groups.^24,33^ In all three countries, disruptions to polio vaccination activities have resulted directly from problems of insecurity and the long-standing absence of health infrastructure in underserved areas and in Afghanistan have been due to outright bans on polio SIAs by local authorities.

**Figure 2.**
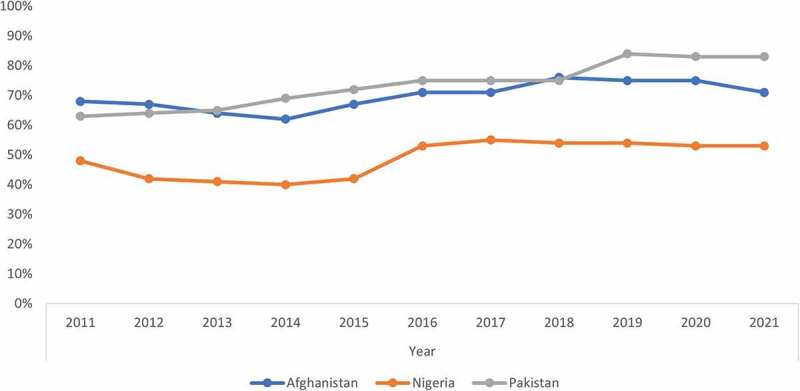
WHO/UNICEF estimates of essential immunization coverage with three doses of oral poliovirus vaccine (OPV) in countries that were endemic for poliovirus transmission during 2011–2021.

Although routine immunization coverage in all three countries has improved over the past decade,^44^ it remains suboptimal, with Nigeria and Pakistan among the top 10 countries with the largest numbers of unvaccinated children.^45^ Comparing national routine immunization coverage estimates with three doses of OPV from 2011 to 2021 (Figure 2), coverage improved from 48% to 53% in Nigeria, 68% to 71% in Afghanistan, and 63% to 83% in Pakistan.^44^ While there is considerable subnational variation in vaccination rates in these countries, coverage is especially poor in areas dogged by conflict and insecurity, where hundreds of thousands of eligible children residing in such areas lack the requisite infrastructure and human resources for essential health service delivery.^46,47^ As a result, their national polio programs tend to be reliant on SIAs to increase immunity in the populations to a level sufficient to interrupt poliovirus transmission. To overcome the problem of inaccessibility in security-compromised areas, several approaches have been adopted.^21^ Meticulous microplanning goes into implementation of each round of SIAs to ensure that as many settlements and neighborhoods as possible that are on the fringes of areas with limited access are covered during vaccination campaigns. This sometimes requires the deployment of mobile teams using various means of transportation across difficult geographic terrain to reach children in these areas.^48,49^ In Nigeria, GIS mapping of settlements and GPS tracking of vaccination teams were incorporated into microplans to enhance the reach and quality of vaccination activities.^50–52^ While these enhancements have helped to improve vaccination coverage in areas that are partially accessible, lot quality assurance sampling (LQAS) surveys and other post-vaccination assessments continue to indicate significant gaps in the quality of vaccination activities in these areas.^41,43,53^

Other initiatives that have been undertaken to increase the reach of vaccination activities to children and families in conflict-affected areas include the establishment of transit-point vaccination teams, synchronization of SIAs between Afghanistan and Pakistan in the light of cross-border movements between both countries, especially among itinerant Pashto-speaking communities, and implementation of health camps, in which a basic package of maternal and child health services is administered alongside polio vaccines.^37,54,55^ These interventions have had varied results and at times more assertive measures have been warranted. In both Pakistan and Nigeria, military operations have liberated certain contested territories, making it possible for vaccination teams to reach children in areas that have been inaccessible for vaccination activities for months to years.^21^ In Borno State, Nigeria, vaccination teams have accessed newly liberated areas when safe or conducted activities in nearby camps for internally displaced persons. They have sometimes been accompanied by armed vigilantes when conducting activities in settlements that were partially under insurgent control. In situations where vaccination teams could not access the area, military health staff have provided vaccination services to eligible children in such areas. Also, in the course of regularly scheduled SIAs, vaccination teams in security compromised areas are often accompanied by police escorts for their safety.^56^ Tragically, both vaccination teams and their police escorts have been victims of targeted attacks in Afghanistan, Pakistan, and Nigeria.^57,58^

GPEI, taking advantage of its political neutrality and interagency structure, continues to work with national governments, local authorities, and humanitarian organizations in security-compromised areas to improve access for vaccination.^59^ Negotiations for access have sometimes resulted in the suspension of bans on vaccination activities and compromises have been reached on the approach adopted for implementation of SIAs. Where the preferred house-to-house vaccination campaigns are not feasible, negotiations with local authorities and anti-government elements have sometimes allowed for campaigns to take place at designated sites; for example, the site-to-site approach adopted in many parts of Afghanistan since 2019.^38,41^ These approaches risk leaving large numbers of children unreached, thus sustaining a pool of susceptible children that maintain the chain of endemic or reestablished transmission of wild poliovirus and allow for new emergences of circulating vaccine-derived polioviruses.

### Outbreak-affected countries (Figure 1)

Outbreaks of both wild poliovirus and, more commonly, vaccine-derived polioviruses continue to create setbacks for polio eradication efforts in areas that have been previously certified as polio-free. In settings of conflict, these outbreaks are often more difficult to control owing to delays in detection and challenges with implementing vaccination activities. For illustrative purposes, outbreaks of WPV that occurred in Somalia and Syria during 2013–2014^14,60^ and subsequent cVDPV2 outbreaks that began in 2017^28–30^ provide instructive examples.

Somalia, a country embroiled in protracted conflict since the fall of its central government in 1991, experienced an outbreak of WPV1 during 2013–2014, with 199 cases being reported.^60,61^ In response to the outbreak, which occurred following importation of WPV1 from Nigeria, multiple rounds of vaccination activities were conducted, including up to 10 rounds of OPV SIAs in 2013 alone.^62^ Complicating the response activities was a ban on polio vaccination activities imposed by anti-government elements in several provinces in the south and central zones of the country, leaving more than 500,000 children under 5 y of age unvaccinated. To get around the problem of inaccessibility resulting from the ban, transit-point vaccination teams were established in districts neighboring inaccessible areas and special, targeted vaccination activities were implemented in areas that became newly accessible during the course of outbreak response activities.^60–63^ Elsewhere in the country, different approaches were used to increase the reach and coverage attained during vaccination campaigns, including expanding the target population from under 5 y to under 15 y and all age groups during some of the campaigns.^62^ These initiatives helped to successfully bring the outbreak under control by August 2014, approximately 16 months after the outbreak was reported in May 2013.^61,64^

A subsequent circulating vaccine-derived poliovirus type 2 outbreak in Somalia,^30^ detected in October 2017, has proven more difficult to control. Despite multiple sequential rounds of mOPV2 vaccination activities, in line with outbreak response guidelines, there remains persistent, low-level transmission of cVDPV2 in Somalia, largely driven by contiguous pockets of inaccessibility that have left hundreds of thousands of eligible children unvaccinated. A similar scenario exists in the conflict-affected areas of southeastern Democratic Republic of Congo, where cVDPV2 circulation has remained recalcitrant, with new emergences often negating the gains attained from control of prior circulating strains of the virus.^31,32^ These scenarios are consistent with what is seen in settings of chronically low routine immunization coverage; immunization coverage in Somalia overall has been <50% for much of the past decade.^62^

Despite a decade-long civil war, Syria has successfully interrupted two major polio outbreaks within months of their identification.^29^ The first, an outbreak of wild poliovirus that was first identified among a cluster of acute flaccid paralysis cases in October 2013, was controlled following a large-scale multinational vaccination drive that involved eight countries in the Middle East region. Although the outbreak spread from Syria to neighboring Iraq, it was effectively contained by April 2014, when the last case of two cases identified in Iraq was reported, the last of 36 cases in Syria having been reported in January 2014. Outbreak response vaccination campaigns in Syria and other countries in the Middle East region continued into 2015, using a combination of bivalent and trivalent OPV for response vaccination activities. Additionally, cross-border vaccination activities were conducted across Syria’s borders with Lebanon, Jordan, Iraq, and Turkey. In these countries, special vaccination activities targeting refugee populations in camps and settlements were also conducted.^14,29^

In 2017, 3 y after the WPV outbreak, Syria experienced a large outbreak of cVDPV2, with 74 cases being reported, all but three of which were from the governorate of Deir-er-Zor.^28,29^ Unlike the ongoing cVDPV2 outbreak in Somalia, where transmission has been sustained over a 5-y period after discovery, the outbreak in Syria was successfully controlled within 4 months of its identification. Three rounds of mOPV2 vaccination activities were implemented in Deir-er-Zor, Raqqa, and parts of Homs governorate, with coverage averaging approximately 80%. Response activities were less expansive compared with the WPV outbreak and were more targeted to areas with evidence of virus circulation and displaced populations from the outbreak zone. Active armed fighting in Deir-er-Zor and evolving political control of the region did not prevent implementation of response vaccination activities to control the outbreak.^29^ Important factors in ensuring cessation of the outbreak within months of its identification were the successful implementation of response vaccination activities coupled with the narrowing of the pool of susceptibles to mostly children aged <2 y due to the large-scale 2013‒2014 response SIAs to the WPV outbreak.^28,29^ Historically good coverage prior to the war, the ability to conduct response activities in areas of insurgency in the midst of active combat and favorable community attitudes toward vaccination proved to be crucial factors in the successful responses.^29^ In contrast, an active outbreak of cVDPV2 in war-torn Yemen^35,65-68^ has proven substantially more difficult to control owing to the inability to implement outbreak response SIAs in several areas with virus circulation. The occupying authorities in these areas have denied access for SIAs. Vaccination response activities in many parts of Yemen have mostly been limited to the delivery of polio vaccines as part of an integrated package of health services that occupying authorities have permitted, an approach that allows for the vaccination of children in areas that are otherwise inaccessible for polio SIAs. However, with more assertive house-to-house immunization denied, this approach is often insufficient as a stand-alone to achieve adequate coverage to stop the outbreak.

## Conclusions

Conflict has been and remains a major obstacle to the eradication of polio.^69^ Endemic transmission of WPV continues in two countries, Afghanistan and Pakistan,^70^ that are significantly affected by protracted conflict and insecurity. Further, outbreaks of vaccine-derived poliovirus continue to occur in conflict-affected geographies, especially in war-torn countries, Somalia and Yemen being notable examples.^68,71^ Not only does conflict interfere with poliovirus surveillance activities, but it also often leaves hundreds of thousands of children inaccessible for routine and supplementary immunization activities.

While implementing polio vaccination activities in conflict-affected geographies has proven challenging, it does not necessarily represent an insurmountable obstacle to eradication efforts in all settings. Within endemic transmission zones in Afghanistan and Pakistan, polio SIAs continue to be implemented, with coverage often hampered by inaccessibility in some areas due to conflict and insecurity.^56,72^ Intermittent bans on polio vaccination activities and sporadic attacks on polio workers and security personnel have further limited the ability to reach and vaccinate children in the most challenging areas for the polio program. Innovative approaches, such as the establishment of transit-point vaccination teams in Afghanistan and community-based vaccination teams in Pakistan, have been used to mitigate the problem of access with varied results.^38,54^ Negotiations for access have led to lifting of bans but sometimes led to less desirable compromises such as substituting the less effective site-to-site vaccination campaigns for the preferred house-to-house vaccination strategy.^38,41^ Prioritizing trust-building activities through community engagement and responsiveness to other emergent health needs within these areas would be pertinent to finding sustainable solutions to the problem of access.

Although Nigeria interrupted wild poliovirus transmission in 2016,^33^ it continues to be dogged by outbreaks of vaccine-derived polioviruses,^68^ especially in areas of the country affected by insecurity from armed banditry and more prone to vaccine resistance. This continues to be the case despite innovative approaches adopted by the polio program. Elsewhere on the continent of Africa, Somalia faces a protracted outbreak of cVDPV2 that has gone on for more than 5 y after declaration,^30,68^ driven by inaccessibility due to bans on polio vaccination activities by anti-government elements. The situation is even more dire in Yemen, where more than 200 children have been paralyzed due to an ongoing cVDPV2 outbreak, which has grown in intensity partly due to the inability to conduct large-scale response vaccination.^65–68^ Syria, having successfully controlled large outbreaks of WPV1 and cVDPV2 despite an ongoing civil war,^14,28,29^ provides a model approach that offers a glimmer of hope that conflict can be overcome as an obstacle to successful polio response activities.

To surmount the challenge of conflict and the attendant inaccessibility of eligible children for vaccination activities, the polio program needs to adopt a multi-pronged approach reflecting the local context that recognizes the limitations of working in these settings and adapting accordingly. Establishment of community-based vaccination programs, where feasible, would provide an avenue to actively engage resistant communities and build the necessary trust needed for polio vaccinators to operate in areas that would otherwise be treacherous. Wherever possible, polio vaccination activities should be integrated with the delivery of other essential health services that meet the felt needs of communities that are affected by conflict and insecurity.

Within the broader context of war and active combat, GPEI can continue to leverage its influence and political neutrality partnering with humanitarian organizations for negotiations for access and helping to create humanitarian corridors and days of tranquility for the delivery of polio vaccines and other health services. Even with these initiatives, it is likely that conflict will continue to pose a significant threat to polio eradication efforts as long as large pockets of susceptible children remain inaccessible for vaccination activities due to insecurity.
